# Influence of denture surface roughness and host factors on dental calculi formation on dentures: a cross-sectional study

**DOI:** 10.1186/s12903-018-0543-1

**Published:** 2018-05-04

**Authors:** Keisuke Matsumura, Yuji Sato, Noboru Kitagawa, Toshiharu Shichita, Daisuke Kawata, Mariko Ishikawa

**Affiliations:** Department of Geriatric Dentistry, Showa University, School of Dentistry, 2-1-1 kitasenzoku Ota Ward, Tokyo, 145-8515 Japan

**Keywords:** Dental calculi on dentures, Denture surface roughness, Denture management, Denture usage

## Abstract

**Background:**

Dental calculi formation on dentures can worsen the oral cavity environment by complicating oral hygiene. However, few studies have investigated the effect of how patients use and manage their dentures, denture surface roughness, and host factors such as oral cavity dryness and saliva properties on denture cleanliness and denture dental calculi formation. Accordingly, we conducted the present survey to evaluate these factors to clarify the strength of the influence of each factor.

**Methods:**

We enrolled 53 patients who had used dentures for at least 3 months and used a dental prosthesis that covered at least the six front teeth including the left and right mandibular canines. After staining the dentures, we divided the participants into a group that was positive for dental calculi (DCP group) and a group that was negative for dental calculi (DCN group). After removing all the stains, we evaluated the surface roughness of the dentures. A questionnaire was used to survey how the participants used and managed their dentures. Oral cavity dryness was evaluated, and resting saliva samples were collected to assess saliva properties. Correlations between the presence or absence of dental calculi and denture use and management were evaluated using a chi-square test. Correlations with denture surface roughness, oral cavity dryness, and saliva properties were evaluated using the Mann–Whitney U test. Correlations between the presence or absence of dental calculi and all factors were analyzed using multivariate analysis (quantification II).

**Results:**

Surface roughness was significantly greater in the DCP group (*p* < 0.01), and the DCP group members wore their dentures during sleep significantly more often and used a denture cleaner when storing their dentures significantly less often (both *p* < 0.01). No significant differences were observed for oral cavity dryness or saliva properties. The multivariate analysis showed significant correlations of dental calculi formation with denture surface roughness and items related to denture use and management, but not for oral cavity dryness or saliva properties.

**Conclusions:**

Our findings indicate that dental calculi formation is influenced by how dentures are used and managed and by denture surface roughness, but not by oral cavity dryness and saliva properties.

## Background

The progressive increase in average lifespan, especially in recent years, has resulted in Japan becoming a “super-aged” society, with 26.7%, or more than one in four people, being 65 years of age or older. As the percentage of elderly people increases, the number of people who use dentures is expected to increase as well.

The adhesion of plaque to dentures may worsen a patient’s oral environment or general health. Denture plaque adheres more easily to rough denture surfaces, and dental calculi can form when plaque reacts with saliva components. Dental calculi adhere strongly to denture surfaces, making home care difficult and creating new areas for dental plaque formation. This can worsen oral hygiene [1, 2] and adversely affect patients’ health by putting them at risk for infections such as pneumonia, which can occur when bacteria enter the lungs along with saliva during silent aspiration [3–5].

Studies investigating the dental calculi formation process have shown that calcium deposits occur from a few hours to 3 days after the appearance of denture plaque [6]. Thus, patients must be instructed how to use and manage their dentures to prevent the long-term adhesion of denture plaque.

The composition of dental calculi resembles that of supragingival calculi that form on natural teeth. Moreover, since saliva components are necessary for dental calculi formation, variations in the oral environment are thought to affect this process [7].

Previous studies have described the relationships between the adhesion of plaque to dentures and factors such as denture surface roughness, methods of denture usage and management, oral cavity dryness, and saliva properties. However, how these factors relate to dental calculi adhesion and how strongly these various factors affect dental calculi formation remain unclear. Therefore, in the present study, we aimed to test the null hypothesis that there is no difference in the degree of influence of these factors on dental calculi formation.

## Methods

This cross-sectional study was approved by the Showa University School of Dentistry ethics screening committee (No. 2015-015). Because dental calculi often form on the polished lingual surface of mandibular dentures, we evaluated only the dental calculi formed in this area. To reduce variation in the data, a single practitioner performed all dental calculi identification.

### Participants

The participants were denture users who visited the Department of Geriatric Dentistry at the Showa University School of Dentistry, had no limb disabilities, and managed their dentures independently at home. After fully explaining the intent of the study, we obtained consent from 53 people (28 men, 25 women; mean age ± standard deviation 79 ± 7.6 years). The subjects’ dentures were full or partial dentures that covered at least the six front teeth including the left and right mandibular canines. People who were in the midst of tissue conditioning or those who had used dentures for less than 3 months prior to the study were excluded, in order to reduce variability within the subject cohort.

### Data collection

Five items comprising 16 factors were surveyed or measured once during a regular health check-up (which typically occurs every 3 months): presence or absence of dental calculi on dentures, denture surface roughness, methods of denture use and management, oral cavity dryness, and saliva properties. To determine presence or absence of dental calculi, a plaque disclosing solution (Rondells Blue Disclosing Pellets®, DIRECTA, Upplands Väsby, Sweden) was used to stain the dentures. This solution produces different colored stains depending on the stage of plaque formation (within 3 days of formation: red; longer than 3 days: blue). After cleaning with a sponge brush, we separated the participants into groups based on visual inspection of dental calculi on dentures. Those who exhibited blue stains and white calcifications were classified into the DCP group, while those who did not show any staining or calcifications were classified into the DCN group.

Denture surface roughness was calculated as arithmetic mean roughness (Ra). Dentures that exhibited blue stains and white calcifications were cleaned with a solution for removing dental calculi (Quick Denture®, GC, Tokyo, Japan) followed by a disinfectant solution (Laborac D®, SUNDENTAL, Osaka, Japan) for 5 min each in an ultrasonic cleaning bath. A surface roughness meter (Surftest SJ-210®, Mitutoyo, Tokyo, Japan) was used to measure denture surface roughness on 4-mm areas of dental calculi adhesion in the DCP group, while reference locations, specifically the polished denture surfaces of the midline, and left and right canines, were measured in the DCN group. The surface roughness for each denture was calculated as a mean of three measurements.

**Fig. 1:**
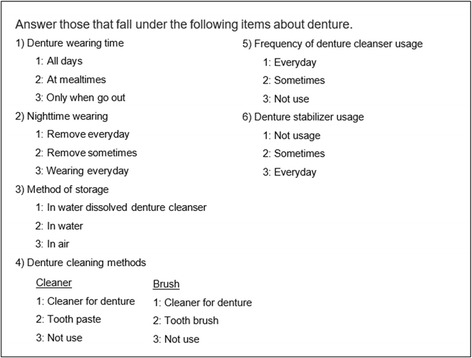
Questionnaire for denture usage / management

Denture use and management was evaluated using a questionnaire based on guidelines for denture cleaning in the clinic and instructions for denture management by dental hygienists from the Japanese Society of Gerodontology [8] (Fig. 1). Interview surveys were conducted after collecting resting saliva.

Oral cavity dryness was evaluated by resting saliva flow rate and oral cavity moisture.

Resting saliva flow rate was measured using the method reported by Fontana et al. [9]. While sitting in a chair, the participant was instructed to swallow the saliva in the mouth at the start of the measurement, and then to incline the head anteriorly in a resting state. The saliva that accumulated in the mouth was collected in a disposable cup, and the saliva accumulated over 5 min was measured to the 0.1 g using a small electric scale (Pocket Scale® CS-240, COSTOM, Tokyo, Japan). Considering that the specific gravity of saliva is 1.003 [10], the amount of saliva secreted per minute was calculated on the basis of the amount of resting saliva collected over 5 min to obtain the resting saliva flow rate (mL/min).

To measure oral cavity moisture, we used an intraoral moisture meter (Mucus®, Life, Saitama, Japan) that uses a sensor to evaluate the static electric capacity of the moisture in the oral mucosal epithelium. With a specialized cover over it, the sensor was pressed perpendicularly onto the measurement sites: buccal mucosa and lingual mucosa. These sites were chosen because they are areas where people often complain of oral cavity dryness and because they are easily accessible [11]. The buccal mucosa measurement was performed at a site approximately 10 mm medial to the angle of the mouth. A finger was used to hold the site lightly from the outside during the measurements. The lingual mucosa measurement was performed on the dorsum of the tongue, approximately 10 mm from the tip. The participants were asked to stick their tongue out during the measurements. The mean value of three measurements for each participant was used in the analysis.

Saliva properties were evaluated based on spinnbarkeit (saliva spinnability), calcium ion (Ca^2+^) concentration, and pH and buffer capacity. The spinnbarkeit of saliva, which expresses the level of viscosity, was measured according to the method reported by Yamagaki et al. [12], using a spinnbarkeit meter (NEVA METER®, Co., Ltd. Ishikawa. Kitakyusyu. Japan). After collection, we placed 60.0 μm^3^ of saliva in a measurement dish with the device set to wet mode. Seven consecutive measurements were made, and the mean of five measurements was used for further analysis after discarding the maximum and minimum values.

Ca^2+^ was measured in 0.25 mL of saliva using a calcium ion meter (Compact Calcium Ion Meter®, B-751, HORIBA, Kyoto, Japan). The mean of three consecutive measurements was used for subsequent analysis. A pH meter (Compact pH meter®, B-712, HORIBA, Kyoto, Japan) was used to measure the pH in 0.25 mL of saliva. Next, an acid-load solution (pH 3, 0.25 mL) was dropped onto and mixed into the saliva to measure buffer capacity. pH and buffer capacity were measured consecutively for each sample. This process was repeated three times and the mean value was used for further analysis.

### Statistical analysis

Presence or absence of dental calculi was compared with the individual measurement and other data items using a chi-square test. Statistical analysis was performed after separating the responses into recommended methods of denture use and management, and other methods. Oral dryness and saliva property items were analyzed using the Mann–Whitney U test. Multivariate analysis (quantification II) was used to examine the correlation between the presence or absence of DC and the factors evaluated.

## Results

Of the 53 participants, 19 were in the DCP group and 34 were in the DCN group. The mean duration of denture use in the DCP group (54 months) was not significantly different from that in the DCN group (29 months). The mean use period was about twice as much as DCP than DCN group, but there was no significant difference between groups. Also, the use period was classified based on the median value, and the use and frequency of use of the denture cleanser during storage were equal. It was suggested that adherence in denture care is subtracted regardless of the use period. The subjects were elderly, which means they were likely taking medications or had reduced physical function; however, none reported experiencing oral dryness or a medication-induced decrease in saliva secretion.

**Fig. 2:**
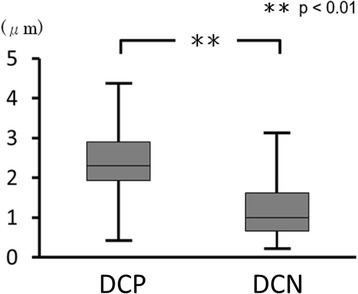
Denture surface roughness (Ra). No significant differences were observed between the DCP and DCN groups

As shown in Fig. 2, the denture surface roughness in the DCP group was significantly higher than that in the DCN group (median 2.3 μm vs 1.0 μm and mean 2.6 μm vs. 1.2 μm, respectively; all *p* < 0.01).

**Fig. 3:**
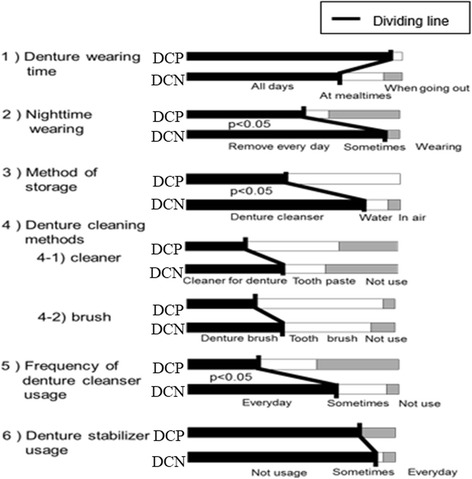
Denture usage / management. Significant differences were observed between the DCP and DCN groups in denture treatment during sleep, denture storage, and the frequency of denture stabilizer usage

With respect to denture usage and management (Fig. 3), the proportion of participants who wore dentures during the day tended to be higher in the DCP group than in the DCN group. Moreover, compared with DCN members, a significantly higher proportion of the DCP members wore their dentures at night (*p* < 0.01) and did not use a denture cleaner at night (p < 0.01).

The proportion of participants who used denture cleaner on their dentures tended to be smaller in the DCP group than in the DCN group, while the proportion who used regular toothpaste tended to be larger in the DCP group. The proportion who used a denture brush tended to be smaller in the DCP group than in the DCN group, while the proportion who used a toothbrush tended to be larger in the DCP group. The proportion of participants who used denture cleaner every day was significantly smaller in the DCP group than in the DCN group (*p* < 0.01).

The proportion of participants who used adhesive regularly was significantly larger in the DCP group than in the DCN group (*p* < 0.01). Some members of the DCP group used adhesive sometimes, but it was used very infrequently in both groups.

**Fig. 4:**
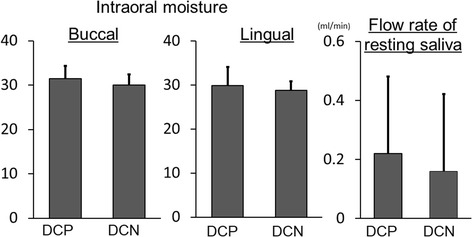
Oral cavity dryness. No significant differences were observed between the DCP and DCN groups

With respect to oral cavity dryness, resting saliva flow rate was slightly higher in the DCP group than in the DCN group, but the difference was not significant (Fig. 4). The DCP group had slightly higher moisture values for both buccal and lingual mucosa, but the differences were not significant (Fig. 4). All values, based on the assessment criteria, were either within or close to the normal range.

**Fig. 5:**
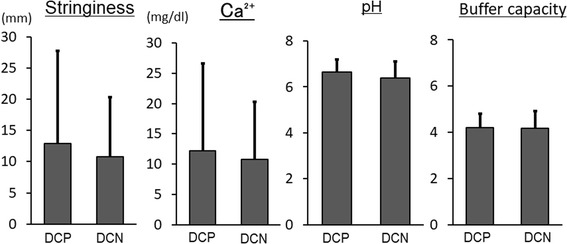
Saliva properties. No significant differences were observed between the DCP and DCN groups

All values for the measured saliva properties were slightly higher in the DCP group, but none of the differences was significant (Fig. 5).

**Fig. 6:**
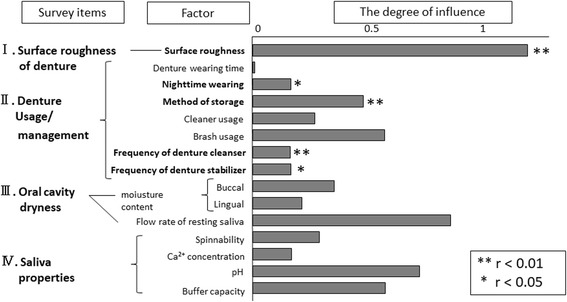
The degree of influence for denture calculi formation. Significant differences were observed regarding how dentures were treated during sleep, how dentures were stored, frequency of denture cleaner use, and frequency of adhesive use

Figure 6 shows the results of the multivariate analysis (quantification II) in the form of a graph that expresses the degree of influence numerically (= category score). The graph shows correlation coefficients for items with significant correlations. Significant correlations were observed between dental calculi adhesion and denture surface roughness, handling during sleep, denture storage methods, frequency of denture cleaner use, and frequency of dental adhesive use. The strongest correlation was between dental calculi adhesion and surface roughness. Significant correlations were not observed for the oral dryness or saliva property items.

## Discussion

The purpose of this study was to clarify the difference of the influence of factors of dental calculi on denture formation. Factors related to dental calculi formation were found to have different degrees of influence, which rejects the null hypothesis. In this cross-sectional study on denture cleaning, participants were selected randomly from among people who used dentures and could manage them independently. The subject dentures were full or partial dentures that covered the mandibular front teeth using a dental plate or artificial teeth. Dental calculi often occur on denture surfaces near the opening of the parotid gland on the maxilla and near the openings of the submandibular and sublingual glands on the mandible. It has been reported that dental calculi are more common on the mandible than on the maxilla [13], so for this study, we chose the lingual side of the mandible to eliminate error due to differences in the dental calculi formation site.

Dental calculi formation is reported to begin with calcification that occurs within 3 days of denture plaque adhesion, with calcification completed by 2 weeks [7]. Because long-term adhesion of denture plaque to the same site can lead to dental calculi formation, it can serve as an indicator of the everyday dental hygiene of denture users. Dental calculi were observed in 36% of the participants surveyed in this study, which is higher than expected and indicates that improvement in dental hygiene is needed.

A relationship between denture surface roughness and bacterial adhesion to resin has been reported [14]. In this study, abrasive paper was used to modify the surface roughness of dentures and the extent of bacterial adhesion was compared between surfaces with different roughness, revealing significantly more bacterial adhesion on rougher surfaces. Further, cleaning dentures with toothpaste containing an abrasive agent can make the surface even rougher [15], indicating the importance of thorough denture polishing during clinic visits and of recommending against the use of abrasive agents for cleaning dentures.

In previous studies [16–19], recommended methods for using and managing dentures included not wearing them at night, using a denture brush, and using denture cleaner frequently. The usefulness of denture brushes was reported in a study that stained dental plaque and then compared the cleaning effects of a denture brush to that of a toothbrush [19], showing that denture brush usage had a greater cleaning effect than toothbrush usage.

In the present study, the use of denture adhesive tended to be more common in the DCP group. Adhesives are used to increase the dentures’ retentive force. However, several studies have found that adhesive usage has adverse effects including deviations of the intercuspal position, alveolar ridge absorption, and other issues [20, 21]. Despite these findings, it has been reported recently that adhesives can be used comfortably if they are used properly under the supervision of a dentist [22–24]. However, it should be noted that the denture adhesive itself has no antimicrobial properties [25]. If used improperly, the surface of adhesive can become rough, and if adhesive is not fully removed, it can provide a surface for the rapid growth of oral microbes. Patients need to be sufficiently warned when they are instructed on using adhesives. These reports are consistent with the results of the present study, demonstrating the importance of appropriate instruction and ensuring patient adherence.

Oral dryness is caused by a decrease in saliva in the oral cavity. This can be caused by reduced saliva secretion, dehydration, or other factors. Reduced saliva secretion decreases self-cleaning functions, which can promote plaque buildup and worsen denture cleanliness [26]. In the present study, we examined resting saliva flow rate and moisture of the oral mucosal epithelium as factors that could potentially influence oral dryness.

Various methods for measuring resting saliva secretion have been reported [27–30]. We selected the spitting method because it is the simplest and is commonly used. The normal resting saliva flow rate is approximately 0.3 mL/min, but is thought to vary widely [31]. Pedersen et al. [32] compared saliva secretion from the submandibular glands in younger people aged 28–39 years with that in elderly people aged 70–90 years. They found that the resting saliva secretion of elderly people was 80% lower than that of younger people. Bertram et al. [33], Brun et al. [34], Mason et al. [35], and Meyer et al. [36] also reported that total saliva secretion decreases with age. However, even though the average age of the participants in the present study was 79 years, their saliva flow was within the normal range.

The device we used for the measurements is useful for objectively evaluating the dryness and moisture content of mucous membranes, but it cannot directly evaluate the amount of saliva secreted [37]. Fukushima et al. [38] reported the following values for oral moisture: normal: ≥29.6, borderline: 28.0–29.5, moderate oral dryness: 25.0–27.9, and severe oral dryness: < 24.9.. The mean values obtained in the present study were all ≥28.0, indicating that while some participants had reduced saliva secretion, none had oral dryness. We found no studies in the literature that examined the relationship between the moisture content of the oral mucosal epithelium and oral cavity cleanliness. However, because low saliva secretion can reduce self-cleaning functions, this may be a useful indicator for making chairside risk predictions. The results of this study suggest that the moisture of the oral mucosal epithelium has little effect on dental calculi formation.

Saliva properties that are thought to affect calculi formation in natural teeth include the presence of plaque buildup with supersaturated calcium phosphate, salivary pH, and salivary proteins. The main component of dental calculi is calcium phosphate [7], and the composition and formation process of dental calculi on dentures resemble those of supragingival calculi formed on natural teeth. Thus, we used the factors affecting dental calculi formation in natural teeth as a reference point when selecting saliva properties to measure as potential factors affecting dental calculi formation in dentures.

No significant differences were observed for any of the saliva properties measured in the present study. This suggests that differences in saliva properties have little influence on dental calculi formation in dentures.

We found no studies that describe the factors affecting spinnbarkeit. That said, if the amount of saliva secreted stays relatively stable, changes in the ratio of serous saliva to mucous saliva could affect spinnbarkeit. As people age, the acinus of their salivary glands atrophy and disappear, a tendency that is particularly marked in the parotid glands. Therefore, the flow of serous saliva may start decreasing at an early stage [39].

Salivary mucin, one of the factors that determine spinnbarkeit in natural teeth, is known to react with calcium and promote the formation of dental calculi. In the present study, higher spinnbarkeit levels were observed in the DCP group, which suggests that spinnbarkeit may contribute to dental calculi formation on dentures.

We also investigated the relationship between Ca^2+^ concentration and salivary pH. Above a critical pH (5.5), free calcium in the oral cavity becomes super saturated with respect to calcium phosphate. Moreover, the ratio of free calcium to total calcium is almost the same, which indicates that an increase occurs as the saliva flow rate rises [28]. Salivary pH is generally constant at approximately 7.0 [40], which makes decalcification of dental calculi that have formed unlikely. Our results showed that Ca^2+^ concentration and pH tended to be higher in the DCP group than in the DCN group. Moreover, participants who wore their dentures all day were constantly in a state in which cleaning and decalcification were less likely, which may have promoted dental calculi formation.

The above results indicate the influence of various factors on dental calculi formation in dentures. They suggest a sequence of events in which plaque adheres to rough denture surfaces, and then calcifies when it is left in place for a long time because of inappropriate denture use or management. Moreover, our investigation of oral cavity dryness and saliva suggests that individual differences could make dental calculi formation more likely. These results provide some evidence indicating that dentists should provide their patients with smooth denture surfaces and instruct them in how to use and manage their dentures appropriately.

We designed a cross-sectional study to confirm the relationships among multiple variables simultaneously. It is important to note this limitation when interpreting the results of this study. The properties and components of saliva fluctuate throughout the day. Therefore, performing an accurate saliva survey requires a large number of subjects and collecting saliva samples over several days. However, the number of samples we could collect was limited because our saliva survey was performed during regular health checks, which included a survey of denture use and management. Therefore, while the results of our investigation of saliva and other factors indicate the importance of denture surface roughness and methods of denture use and management, it does not establish proof. A longitudinal study would be necessary to substantiate the relationships suggested by our findings.

In this study, we examined the lingual polished surfaces of mandibular dentures to obtain uniform measurements. In future studies, we plan to examine maxillary dentures. In addition, by expressing the results of questionnaire surveys and host factors numerically, we hope to create assessment sheets that can help improve the oral cleanliness of denture patients.

## Conclusions

The results of this study indicate that denture surface roughness and the methods of denture use and management significantly affect dental calculi formation. Furthermore, our findings suggest that oral cavity dryness and saliva properties have little influence on dental calculi formation.

## Abbreviations

DCN: Dental calculi negative
DCP: Dental calculi positive
